# Retraction Note: Health-related quality of life among ethnic minority residents in remote Western China: a cross-sectional study

**DOI:** 10.1186/s12889-023-16559-x

**Published:** 2023-08-25

**Authors:** Jiaxin Dong, Xiaoju Li, Rong Fan, Jielin Yang

**Affiliations:** 1 Department of Public Health, Shihezi University School of Medicine, North 2Th Road, Shihezi, 832000 Xinjiang China; 2 Infection Management Department, Dehong Prefecture People’s Hospital, Dehong, 678400 Yunnan China

**Retraction Note: BMC Public Health 23, 638 (2023)**

**https://doi.org/10.1186/s12889-023-15544-8**

The Editor has retracted this article because the authors had not obtained appropriate ethical approval before the recruitment of participants for this study began. None of the authors agree to this retraction.

